# Supplementary figures and images for: Identification of protein signatures for lung cancer subtypes based on BPSO method
Source: PLoS One. 2023 Dec 7;18(12):e0294243. doi: 10.1371/journal.pone.0294243 (PMC10703216; doi:10.1371/journal.pone.0294243)

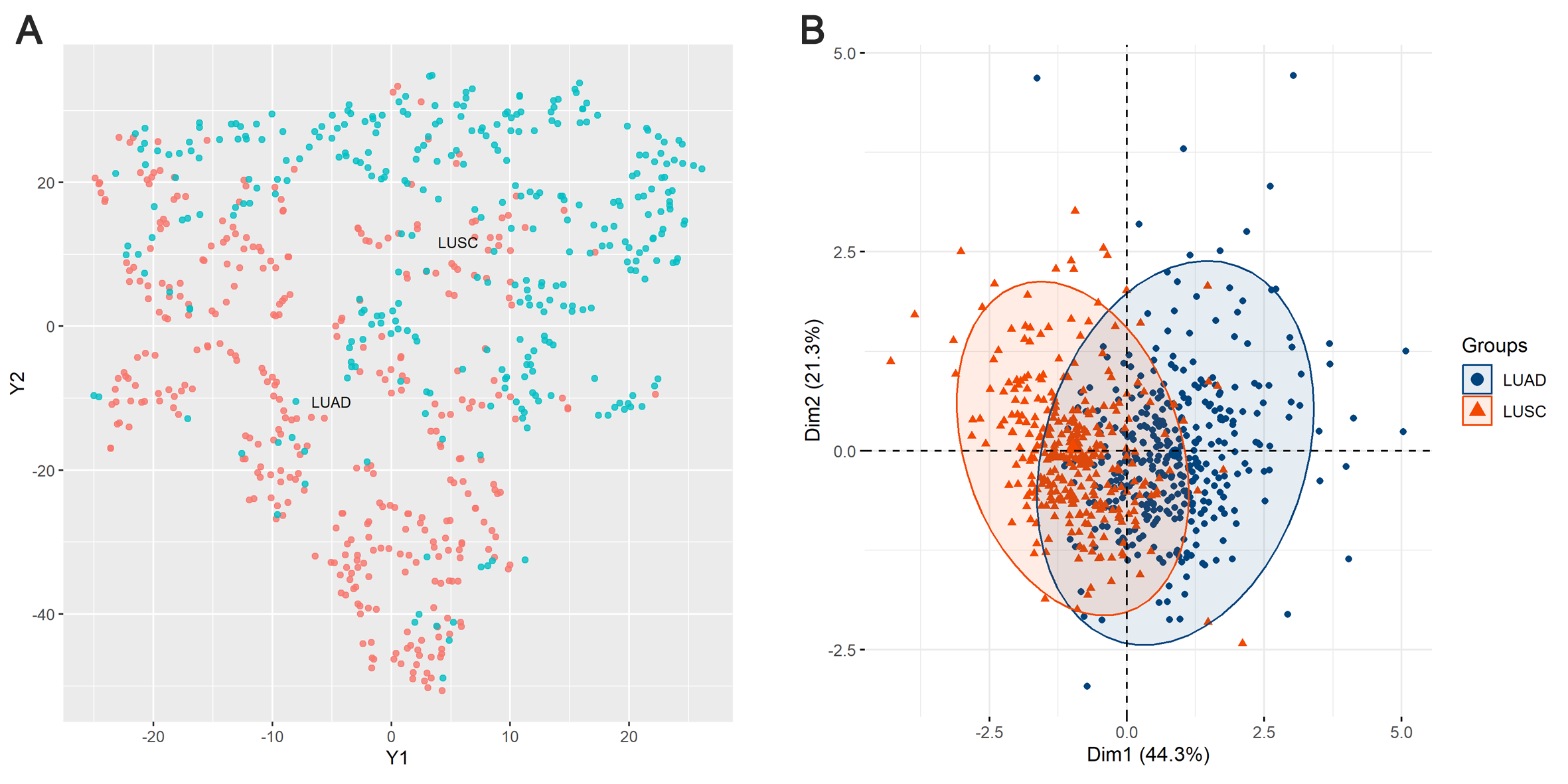

Supplement: S1 Fig — (A) t-SNE showed the clusters of samples based on 4 proteins profiling. (B) PCA showed the clusters of samples based on 4 proteins profiling. (TIF) [file pone.0294243.s001.tif]
