# Supplementary material for: Identification of protein signatures for lung cancer subtypes based on BPSO method
Source: PLoS One. 2023 Dec 7;18(12):e0294243. doi: 10.1371/journal.pone.0294243 (PMC10703216; doi:10.1371/journal.pone.0294243)
Supplement: S1 Table — (DOCX) [file pone.0294243.s002.docx]

**Table S1.** Differential protein analysis between LUAD vs. LUSC

| **Order** | **Proteins** | **LogFC** | **PValue** | **Up/Down** |
| --- | --- | --- | --- | --- |
| 1 | **GAPDH** | 1.43 | 3.30E-29 | Up |
| 2 | **TFRC** | 1.30 | 1.78E-54 | Up |
| 3 | EGFR_pY1068 | -1.10 | 2.25E-14 | Down |
| 4 | NDRG1_pT346 | 0.98 | 1.55E-41 | Up |
| 5 | **IGFBP2** | 0.83 | 2.91E-20 | Up |
| 6 | **INPP4B** | -0.77 | 1.89E-19 | Down |
| 7 | DUSP4 | -0.61 | 1.70E-09 | Down |
| 8 | PAI1 | 0.55 | 5.02E-12 | Up |
| 9 | AKT_pT308 | 0.54 | 1.81E-08 | Up |
| 10 | EPPK1 | 0.52 | 9.05E-08 | Up |

Here showed the 10 most significantly differential proteins between LUAD vs. LUSC, the analysis was performed using “edgeR” package in R.
