# Supplementary material for: Identification of protein signatures for lung cancer subtypes based on BPSO method
Source: PLoS One. 2023 Dec 7;18(12):e0294243. doi: 10.1371/journal.pone.0294243 (PMC10703216; doi:10.1371/journal.pone.0294243)
Supplement: S2 Table — (DOCX) [file pone.0294243.s003.docx]

**Table S2.** Analysis of the 10 selected proteins between LUAD vs. LUSC

| **Proteins** | **RPPA protein abundances (average)** | | | **ROC analysis** | | | |
| --- | --- | --- | --- | --- | --- | --- | --- |
|  | **LUAD** | **LUSC** | **Up/Down** | **Sensitivity** | **Specificity** | **AUC** | **PValue** |
| TFRC | -0.396 | 0.943 | Up | 84.20% | 67.70% | 0.808 | <0.001 |
| CD26 | 0.020 | -0.298 | Down | 73.50% | 65.70% | 0.755 | <0.001 |
| MIG6 | 0.214 | -0.081 | Down | 84.20% | 60.10% | 0.767 | <0.001 |
| GAPDH | -2.336 | -0.898 | Up | 75.10% | 62.40% | 0.739 | <0.001 |
| INPP4B | 0.624 | -0.096 | Down | 73.90% | 61.30% | 0.724 | <0.001 |
| FOXM1 | -0.386 | 0.013 | Up | 71.50% | 61.10% | 0.716 | <0.001 |
| NF2 | -0.122 | 0.272 | Up | 62.50% | 69.60% | 0.693 | <0.001 |
| ACVRL1 | -0.127 | -0.237 | Down | 68.80% | 59.90% | 0.682 | <0.001 |
| IGFBP2 | -0.102 | 0.774 | Up | 56.90% | 74.20% | 0.686 | <0.001 |
| X4EBP1 | -0.176 | 0.275 | Up | 60.90% | 72.40% | 0.698 | <0.001 |
