# Supplementary material for: Identification of protein signatures for lung cancer subtypes based on BPSO method
Source: PLoS One. 2023 Dec 7;18(12):e0294243. doi: 10.1371/journal.pone.0294243 (PMC10703216; doi:10.1371/journal.pone.0294243)
Supplement: Attachment — Submitted filename: Response to Reviewers.docx [file pone.0294243.s004.docx]

**Reviewer #1:** The aim of this article is to identify protein biomarkers that can distinguish LUAD from LUSC, which is essential for personalized treatment regimens. Using the BPSO feature selection method, the authors identified 10 candidate protein biomarkers with good classification performance by t-SNE and PCA algorithm analysis. To explore the causal relationship between these proteins and their association with tumor subtypes, a regulatory network was constructed using the PCStable algorithm. The authors also used Mauman's correlation, Cox proportional hazards models, and Kaplan-Meier curves to verify the biological significance of the candidate proteins. However, I have some suggestions and questions about certain parts of the manuscript.

**We thank the reviewer so much for the positive and valuable comments.**

1. According to what criteria are the authors selected 10 candidate protein features by BPSO algorithm?

**Answer:** We thank the reviewer's valuable and thoughtful concern. Our methodology for obtaining 10 candidate proteins was performed by two steps:

First, we used signal-to-noise ratio (SNR) filter feature selection method to remove these proteins whose SNR value was less than 0.4, and 19 remain proteins were employed for further analysis.

Second, the 10 candidate protein features were automatically extracted from the previous 19 proteins based on a specific parameter set of BPSO. The parameter set has shown in section 3.2 of the modified manuscript.

In addition, the raw data and source code of BPSO we used in this research were uploaded to GitHub, which can be found in <https://github.com/jihanwang/BPSO/>.

2. Whether NF2 and INPP4B have been demonstrated as prognostic markers in lung cancer.

**Answer:** We thank the reviewer's valuable and careful suggestion. After reviewing related literatures, we found no direct research on NF2 and INPP4B as prognostic markers in lung cancer. However, NF2 has been reported as a prognostic marker in other malignant diseases, such as the article "The prognostic significance of BAP1, NF2, and CDKN2A in malignant peritoneal mesothelioma" (https://pubmed.ncbi.nlm.nih.gov/26493618/) and "Current and emerging biomarkers in tumors of the central nervous system: Possible diagnostic, prognostic and therapeutic applications" (https://pubmed.ncbi.nlm.nih.gov/28774835/). Similarly, INPP4B has been reported as a prognostic marker in a variety of cancers, such as "INPP4B As A Prognostic And Diagnostic Marker Regulates Cell Growth Of Pancreatic Cancer Via Activating AKT" (https://pubmed.ncbi.nlm.nih.gov /31632078/)", “INPP4B overexpression is associated with poor clinical outcome and therapy resistance in acute myeloid leukemia" (https://pubmed.ncbi.nlm.nih.gov/25736236/), “Expression of inositol polyphosphate 4-phosphatase type II and the prognosis of oral squamous cell carcinoma” (https://pubmed.ncbi.nlm.nih.gov/32027770/); etc.

The above results also reflect the application value of NF2 and INPP4B as cancer prognostic markers. Regarding their mechanism in lung cancer need to be continued research.

3. The authors have repeatedly described that the feature selection method BPSO is superior to other methods, but the speed, computational cost and robustness of other methods are not compared in the results.

**Answer:** We thank the reviewer's professional concern. First, we need to clarify that in the original article we explained that BPSO has faster computing speed and robustness than PSO. The detailed basis can refer to "A novel binary particle swarm optimization. In 2007 Mediterranean conference on control & automation, pp. 1-6. IEEE, 2007. ".

For computational cost and robustness:

(1) Low Computational Complexity: PSO is known for its simplicity and relatively low computational complexity. It does not involve complex mathematical operations or gradients, making it suitable for solving optimization problems in real-time or with limited computational resources. BPSO is a simplified version of PSO and has lower computational complexity than PSO.

(2) Robustness: Global and Local Search Capabilities: BPSO maintains a balance between exploration and exploitation. It combines both global search (exploration) by adjusting particles' positions based on the collective knowledge of the swarm and local search (exploitation) by considering each particle's personal best. Less Sensitive to Initial Conditions: BPSO is generally less sensitive to initial conditions compared to some other optimization algorithms like gradient descent. This makes it easier to use and more forgiving of initial parameter choices.

To be more rigorous, we also compared the accuracy of BPSO and Random-Froest (RF) from the aspect of classification accuracy. To demonstrate the advantages of BPSO in classification accuracy, here we used the RF feature selection method to obtain the top 10 candidate proteins and compared their classification accuracy based on PCA. The PCA result of RF was as follow. For RF, the PC1 (Dim1) score was 34% and PC2 (Dim2) was 11.2%. For BPSO, the PC1 (Dim1) score was 37.5% and PC2 (Dim2) was 11.8%, which was shown in Figure 1D in the manuscript. The result indicated that BPSO is superior to RF method in lung cancer subtype classification.

**PCA model based on the top 10 candidate proteins by using RF method**

4. The experimental verification of the four proteins as lung cancer biomarkers is insufficient, and it is recommended to add more experiments to verify the effects of the four proteins on lung cancer.

**Answer**: We thank the reviewer’s constructive comments. Indeed, as you suggested, it's necessary to conduct more experiments to verify the effects of the four proteins on lung cancer, to support the findings in this study. It should be noted that our study mainly emphasizes the application of feature selection method in high-dimensional proteomic data. In this regard, we used the BPSO algorithm to classify lung cancer subtypes based on the proteomic data in TCPA, and obtained 10 proteins that better in classifying cancer subtypes.

To further verify the findings, here we analyzed these 10 proteins and their corresponding coding genes, and the results showed that the changes in protein abundance and mRNA expression levels of these 10 proteins were consistent, as shown in the table below. Moreover, PCA based on these 10 genes can clearly distinguish LUAD and LUSC, as shown in the figure below. This part of the results indirectly confirms that the proteins we screened are significantly differentially expressed between the two subgroups, which helps to identify and distinguish the two cancer subtypes. In future research, we will conduct corresponding mechanism and function research.

**Analysis of the 10 selected proteins as well as their coding genes between LUAD vs. LUSC**

| **Proteins** | **RPPA protein abundances (average)** | | | **Genes** | **mRNA expression (average)** | | |
| --- | --- | --- | --- | --- | --- | --- | --- |
|  | **LUAD** | **LUSC** | **Up/Down** |  | **LUAD** | **LUSC** | **Up/Down** |
| TFRC | -0.396 | 0.943 | Up | TFRC | -2.115 | 1.609 | Up |
| CD26 | 0.020 | -0.298 | Down | CD26 | 0.643 | -5.600 | Down |
| MIG6 | 0.214 | -0.081 | Down | ERRFI1 | 0.536 | -1.141 | Down |
| GAPDH | -2.336 | -0.898 | Up | GAPDH | 4.150 | 5.595 | Up |
| INPP4B | 0.624 | -0.096 | Down | INPP4B | 1.530 | -0.565 | Down |
| FOXM1 | -0.386 | 0.013 | Up | FOXM1 | 3.939 | 4.091 | Up |
| NF2 | -0.122 | 0.272 | Up | NF2 | -0.133 | 0.743 | Up |
| ACVRL1 | -0.127 | -0.237 | Down | ACVRL1 | -3.944 | -7.687 | Down |
| IGFBP2 | -0.102 | 0.774 | Up | IGFBP2 | 0.652 | 3.384 | Up |
| X4EBP1 | -0.176 | 0.275 | Up | EIF4EBP1 | 2.119 | 5.456 | Up |

Note: The values of RPPA protein abundances for each protein were derived from the TCPA database. The values of mRNA expression for each gene (the corresponding protein-coding gene) were derived from the cBioPortal database (<https://www.cbioportal.org/>), the LUAD and LUSC cohorts belongs to the TCGA PanCancer Atlas. The values of mRNA expression are mRNA expression z-scores relative to normal samples (log RNA Seq V2 RSEM).

**PCA model based on the expression of 10 corresponding protein-coding genes**

**Reviewer #2:** The overall idea of identifying protein markers in order to differentiate lung adenocarcinoma and squamous cell carcinoma is beneficial and important. The results were obtained using the protein expression dataset for lung cancer from the public TCPA database.

**We thank the reviewer so much for the positive remarks and careful review.**

Major concerns are in the following points:

1. In the introduction and in the discussion, the authors claimed they “identify candidate protein biomarkers that outperformed the original features in classifying the two major lung cancer subtypes." and “The classification performance of the 10 protein signatures outperformed that of all proteins, as evidenced by t-SNE and PCA.”

a. Both t-SNE and PCA are graphical visualization approaches, but no quantitative measurements (e.g. sensitivity, specificity, AUC) were used to evaluate the classification accuracy for adenocarcinoma vs squamous cell carcinoma.

**Answer**: We thank the reviewer’s careful and insightful suggestions. We have performed ROC analysis using Medcalc software and evaluated the sensitivities, specificities, AUC and p-values of the 10 selected proteins. The following table showed the detailed results and has added as supplementary materials (Table S2 in the revised manuscript).

**Table S2.** Analysis of the 10 selected proteins between LUAD vs. LUSC

| **Proteins** | **RPPA protein abundances (average)** | | | **ROC analysis** | | | |
| --- | --- | --- | --- | --- | --- | --- | --- |
|  | **LUAD** | **LUSC** | **Up/Down** | **Sensitivity** | **Specificity** | **AUC** | **PValue** |
| TFRC | -0.396 | 0.943 | Up | 84.20% | 67.70% | 0.808 | <0.001 |
| CD26 | 0.020 | -0.298 | Down | 73.50% | 65.70% | 0.755 | <0.001 |
| MIG6 | 0.214 | -0.081 | Down | 84.20% | 60.10% | 0.767 | <0.001 |
| GAPDH | -2.336 | -0.898 | Up | 75.10% | 62.40% | 0.739 | <0.001 |
| INPP4B | 0.624 | -0.096 | Down | 73.90% | 61.30% | 0.724 | <0.001 |
| FOXM1 | -0.386 | 0.013 | Up | 71.50% | 61.10% | 0.716 | <0.001 |
| NF2 | -0.122 | 0.272 | Up | 62.50% | 69.60% | 0.693 | <0.001 |
| ACVRL1 | -0.127 | -0.237 | Down | 68.80% | 59.90% | 0.682 | <0.001 |
| IGFBP2 | -0.102 | 0.774 | Up | 56.90% | 74.20% | 0.686 | <0.001 |
| X4EBP1 | -0.176 | 0.275 | Up | 60.90% | 72.40% | 0.698 | <0.001 |

b. The authors didn’t compare the classification accuracy of their method to the prior outstanding approaches. For instance, the prior study (Ref. 8: DOI: 10.1038/modpathol.2011.92) demonstrated that “a two-marker panel of TTF-1/p63 is sufficient for subtyping of the majority of tumors as adenocarcinomas vs squamous cell carcinoma”. Did the authors compare the predictive performance of the selected 10 or 4 protein signatures with the two-marker panel of TTF-1/p63?

**Answer**: We thank the reviewer for the valuable and remarkable suggestions. Indeed, the prior study (Ref. 8: DOI: 10.1038/modpathol.2011.92) is very inspiring and helpful to us. It should be noted that: we first downloaded all protein expression data in TCPA (including a total of 258 proteins), and then preprocessed the ataset. During the preprocessing, we removed some proteins with values of "NA". Unfortunately, the two proteins, TTF-1 and p63, were removed during the preprocessing, since the samples had missing values of TTF-1 and p63. Specifically, among 362 LUAD samples, TTF-1 and p63 had missing values in 128 samples; among 325 LUSC samples, TTF-1 and p63 had missing values in 133 samples, the raw data with missing values can be found in https://github.com/jihanwang/BPSO/. In response to your concerns, we tried to analyze the remaining samples without missing values, including 234 cases of LUAD and 192 cases of LUSC. ROC analysis showed that the Sensitivity, Specificity, and AUC of TTF1 in differentiating the two subtypes were 90.1%, 80.3%, and 0.880 respectively; the Sensitivity, Specificity, and AUC of p63 in differentiating the two subtypes were 85.4% and 45.3%, 0.671 respectively. The above results suggest that these two proteins have certain values for distinguishing LUAD and LUSC, especially TTF1. By considering your question, we have also thought about some issues related to data preprocessing. How to exclude meaningless samples or features and retain valuable samples or features as much as possible requires further thinking. We also included this point in the limitations of this study. In summary, our study provides some complementary biotargets for lung cancer research.

We thank you again for your valuable feedback!

c. Moreover, the proposed BPSO algorithm is a feature selection approach, the classification accuracy by using the selected features should be validated by using cross-validation and/or on other independent datasets.

**Answer**: We thank the reviewer’s professional feedback.

First, we have uploaded our BPSO source code to the GitHub website, which can be found in <https://github.com/jihanwang/BPSO/>. The BPSO algorithm is a packaging feature selection method that obtains the optimal classification feature subset through continuous iteration. In each interation process, the sample data set will be randomly allocated into a training set and a test set, and the feature subset obtained in each iteration will be used to test its classification accuracy, which is essentially a cross-validation. Therefore, the feature subset obtained by this algorithm has strong generalization.

Second, to further verify the findings, here we analyzed these 10 proteins and their corresponding coding genes, and the results showed that the changes in protein abundance and mRNA expression levels of these 10 proteins were consistent, as shown in the table below. Moreover, PCA based on these 10 genes can clearly distinguish LUAD and LUSC, as shown in the figure below. This part of the results indirectly confirms that the proteins we screened are significantly differentially expressed between the two groups, which helps to identify and distinguish the two cancer subtypes. In future research, we will conduct corresponding mechanism and function research.

**Analysis of the 10 selected proteins as well as their coding genes between LUAD vs. LUSC**

| **Proteins** | **RPPA protein abundances (average)** | | | **Genes** | **mRNA expression (average)** | | |
| --- | --- | --- | --- | --- | --- | --- | --- |
|  | **LUAD** | **LUSC** | **Up/Down** |  | **LUAD** | **LUSC** | **Up/Down** |
| TFRC | -0.396 | 0.943 | Up | TFRC | -2.115 | 1.609 | Up |
| CD26 | 0.020 | -0.298 | Down | CD26 | 0.643 | -5.600 | Down |
| MIG6 | 0.214 | -0.081 | Down | ERRFI1 | 0.536 | -1.141 | Down |
| GAPDH | -2.336 | -0.898 | Up | GAPDH | 4.150 | 5.595 | Up |
| INPP4B | 0.624 | -0.096 | Down | INPP4B | 1.530 | -0.565 | Down |
| FOXM1 | -0.386 | 0.013 | Up | FOXM1 | 3.939 | 4.091 | Up |
| NF2 | -0.122 | 0.272 | Up | NF2 | -0.133 | 0.743 | Up |
| ACVRL1 | -0.127 | -0.237 | Down | ACVRL1 | -3.944 | -7.687 | Down |
| IGFBP2 | -0.102 | 0.774 | Up | IGFBP2 | 0.652 | 3.384 | Up |
| X4EBP1 | -0.176 | 0.275 | Up | EIF4EBP1 | 2.119 | 5.456 | Up |

Note: The values of RPPA protein abundances for each protein were derived from the TCPA database. The values of mRNA expression for each gene (the corresponding protein-coding gene) were derived from the cBioPortal database (<https://www.cbioportal.org/>), the LUAD and LUSC cohorts belongs to the TCGA PanCancer Atlas. The values of mRNA expression are mRNA expression z-scores relative to normal samples (log RNA Seq V2 RSEM).

**PCA model based on the expression of 10 corresponding protein-coding genes**

2. In Figure 3, the network analysis identified 4 proteins “directly linked” to the classification of adenocarcinoma vs squamous cell carcinoma. It doesn’t currently make scientific sense how the four proteins will directly impact the subtype classification. Please discuss this, otherwise it should be discussed as a limitation.

**Answer**: we thank the reviewer for bringing this to ensure the accuracy and clarity of our findings in the revised version of the paper.

First, the Fig S1 has shown that the four proteins, including MIG6, CD26, NF2, and INPP4B, showed significant discriminatory power between the subtypes based on their abundance profiles.

Second, we acknowledge your concern regarding the term "directly linked" in Figure 3. We appreciate your astute observation. Additionally, we acknowledge that while Bayesian network is a valuable causal exploratory tool and provides a graphical depiction of the inferred probabilistic dependencies based on the available data, it has inherent limitations and should be interpreted with caution.

Third, we will try to experimentally validate and further elucidate the roles of these proteins in subtype classification in our future research.

Final, we have added the limitation you concern in the discussion.

Minor:

Legends or contents in some figures are too small, or low resolution (e.g. Figures 1 and 5).

**Answer**: We thank the reviewer’s careful and valuable feedback. All images in this study were generated based on a resolution of 300dpi. The reason for the low resolution of picture as you mentioned may be that some distortion occurs when the system embeds the picture into word.
